# Marek’s Disease Virus Activates the PI3K/Akt Pathway Through Interaction of Its Protein Meq With the P85 Subunit of PI3K to Promote Viral Replication

**DOI:** 10.3389/fmicb.2018.02547

**Published:** 2018-10-23

**Authors:** Huimin Li, Jiaojiao Zhu, Minyi He, Qiong Luo, Fan Liu, Ruiai Chen

**Affiliations:** College of Veterinary Medicine, South China Agricultural University, Guangzhou, China

**Keywords:** Marek’s disease virus, PI3K/Akt signaling pathway, virus replication, apoptosis, protein interaction

## Abstract

It is known that viruses can active the phosphatidylinositol 3-kinase (PI3K)/Akt signaling pathway in host cells to support cell survival and viral replication; however, the role of PI3K/Akt signaling in the pathogenic mechanisms induced by Marek’s disease virus (MDV) which causes a neoplastic Marek’s disease in poultry, remains unknown. In this study, we showed that MDV activated the PI3K/Akt pathway in chicken embryo fibroblasts (CEFs) at the early phase of infection, whereas treatment with a PI3K inhibitor LY294002 prior to MDV infection decreased viral replication and DNA synthesis. Flow cytometry analysis showed that inhibition of the PI3K/Akt pathway could significantly increase apoptosis in MDV-infected host cells, indicating that activation of PI3K/Akt signaling could facilitate viral replication through support of cell survival during infection. Evaluation of the underlying molecular mechanism by co-immunoprecipitation and laser confocal microscopy revealed that a viral protein Meq interacted with both p85α and p85β regulatory subunits of PI3K and could induce PI3K/Akt signaling in Meq-overexpressing chicken fibroblasts. Our results showed, for the first time, that MDV activated PI3K/Akt signaling in host cells through interaction of its Meq protein with the regulatory p85 subunit of PI3K to delay cell apoptosis and promote viral replication. This study provides clues for further studies of the molecular mechanisms underlying MDV infection and pathogenicity for the host.

## Introduction

Marek’s disease (MD) is caused by Marek’s disease virus (MDV), a highly oncogenic cell-associated α-herpesvirus inducing T-cell lymphoma, immunosuppression, and neurological disorders in poultry (Jarosinski et al., 2006). MDV is highly contagious and the infection can cause significant economic losses for the poultry industry throughout the world (Biggs and Nair, 2012). Although vaccination has been shown to be an effective measure to prevent and control MD, it cannot completely stop disease outbreaks recorded worldwide (Cui et al., 2016; Lopez-Osorio et al., 2017; Abd-Ellatieff et al., 2018), because the virus can still replicate in vaccinated birds, which can shed infectious MDV, thus spreading the disease. Furthermore, MDV was shown to gradually evolveunder environmental pressure, which resulted in the emergence of new pathogenic subtypes and increase of virulence (Witter, 1997; Davison and Nair, 2005). Therefore, it is necessary to perform comprehensive investigation of the molecular mechanisms underlying the interaction of MDV with host cells in order to determine the critical points to be used as targets for the development of new vaccines and antiviral drugs.

The Meq oncogene plays a key role in MDV-induced lymphomagenesis and as evidenced by the lack of carcinogenicity of recombinant Meq-deficient MDV particles in chickens (Brown et al., 2009; Silva et al., 2010). Meq has an N-terminal basic DNA-binding domain adjacent to a leucine zipper (Jones et al., 1992), suggesting its relatedness to the Jun/Fos family of transcription factors. Through the leucine zipper, Meq can form homodimers or heterodimers with the c-Jun oncoprotein (Qian et al., 1996; Kung et al., 2001) and induce the expression of anti-apoptotic genes associated with oncogenic transformation of host cells (Jarosinski et al., 2006). Furthermore, Meq interaction with carboxyl-terminal-binding protein (CtBP) via the PXDLS motif is critical for tumorigenesis but not for MDV replication in chickens (Brown et al., 2006). MDV can also interact with heat shock protein 70 (hsp70) (Zhao et al., 2009) and a tumor suppressor protein p53 (Deng et al., 2010), suggesting that these proteins are potentially involved in MD pathogenesis. According to the results of ChIP-seq analysis, 549 genes are downregulated and 236 are upregulated in Meq-overexpressing DF-1 chicken fibroblast cell line (Subramaniam et al., 2013), indicating that many more genes are potentially targeted by Meq in host cells and these interactions should be investigated.

The phosphatidylinositol 3-kinase (PI3K)/Akt signaling pathway is involved in the regulation of many key cellular processes, including growth, proliferation, survival, migration, metabolism, and apoptosis (Manning and Toker, 2017). Based on substrate preference and sequence homology, PI3Ks are divided into three classes; among them, class IA enzymes are heterodimers composed of a regulatory subunit (p85α, p55α, p50α, p85β, or p55γ) and a catalytic subunit (p110α, p110β, or p110δ) (Engelman et al., 2006). When PI3K is activated via G protein-coupled receptors (GPCR) or receptor tyrosine kinases (RTKs), the p110 subunit generates phosphatidylinositol 3,4,5-triphosphate (PIP3) from phosphatidylinositol 3,4-bisphosphate (PIP2), whereas p85 is responsible for the interaction of PI3K with the upstream signaling proteins through SH2 and SH3 domains (Carpenter et al., 1993; Street et al., 2004). The produced PIP3 binds to a serine/threonine protein kinase Akt, which is recruited to the plasma membrane (Vanhaesebroeck et al., 2010) where it is phosphorylated at Thr308 and Ser473 by its activating kinases. In turn, the phosphorylated Akt activate or deactivate its numerous targets such as glycogen synthase kinase-3 (GSK-3), p53, NF-κB, and FOXO1, thus playing a major role in modulating diverse downstream signaling pathways associated with cell proliferation, migration, differentiation, and apoptosis (De Luca et al., 2012).

It is known that various viruses can active the PI3K/Akt pathway in order to block cell apoptosis and facilitate virus replication, assembly, and release. For example, the NS1 protein of influenza A virus interacts with the p85β subunit of PI3K, which results in the activation of PI3K/Akt signaling, inhibition of cell apoptosis, and promotion of virus replication (Ehrhardt et al., 2007; Shin et al., 2007a,b), whereas the VP5 protein of infectious bursal disease virus binds to the p85α subunit, which also suppresses apoptosis of host cells and benefits virus growth (Wei et al., 2011). K1, a transmembrane glycoprotein encoded by KSHV contains a ITAM motif which interacts with SH2 domain-containing of p85 subunit to activate PI3K/AKT/mTOR signaling (Bhatt and Damania, 2013). A recent study shows that LMP1, the principal oncoprotein of Epstein-Barr virus (EBV) causing nasopharyngeal carcinoma, contains a PXXP motif which can interact with the p85 subunit of PI3K and activate PI3K/Akt signaling (Wang et al., 2017). On the other hand, PI3K/Akt activation propitious for the replication of these viruses, suppresses that of mammalian reoviruses, hepatitis B virus, and porcine epidemic diarrhea virus (Guo et al., 2007; Tian et al., 2015; Kong et al., 2016). However, to date, there are no data regarding the relationship between MDV infection and PI3K/Akt activation in host cells.

In this study, we investigated the association among MDV infection, PI3K/Akt signaling, and host cell apoptosis. Our results indicate that MDV promoted Akt phosphorylation in a PI3K-dependent manner at the early stage of infection, whereas LY294002, a specific inhibitor of PI3K, suppressed MDV replication and induces apoptosis of chicken embryo fibroblasts (CEFs). By addressing the underlying molecular mechanisms, we found that the Meq protein of MDV interacted with both p85α and p85β subunits of PI3K and activated the PI3K/Akt signaling. These findings suggest that MDV, through Meq, activates the PI3K/Akt pathway to prevent host cell apoptosis and support virus replication.

## Materials and Methods

### Cells and Viruses

Primary CEFs were obtained from 10-day-old specific-pathogen free (SPF)-embryonated chicken eggs (Guangdong Wens Dahuanong Biotechnology Co., Ltd. Guangzhou, China). Chicken fibroblast cell line DF-1 was purchased from the American Type Culture Collection (ATCC, Manassas, VA, United States). CEF and DF-1 cells were grown in Dulbecco’s modified Eagle medium (DMEM; Gibco, Thermo Fisher Scientific, Waltham, MA, United States) with 10% heat-inactivated fetal bovine serum (FBS), 100 U/mL penicillin, and 100 μg/mL streptomycin (Gibco) at 37°C with 5% CO2. For the experiments, cells were seeded overnight and used at approximately 80% confluence.

MDV strains LZ1309 (very virulent MDV, vvMDV), Md5 (vvMDV), and 814 (mild MDV, mMDV) were obtained from South China Agricultural University (Guangzhou, China).

### Virus Infection

Cells were grown overnight at 37°C with 5% CO2 until reached approximately 80% confluence. After starvation in serum-free medium for 1 h, cells were washed with PBS and incubated with MDV diluted in DMEM to the multiplicity of infection (MOI) of 0.1 for different times at 37°C. After the inoculum was aspirated, cells were grown in DMEM supplemented with 10% FBS, 100 U/ml penicillin, and 0.1 mg/ml streptomycin.

### Replication of MDV

The MDV replication *in vitro* was measured by counting the number of plaques in the CEFs at various time points. Briefly, 100 plaque forming units (pfu) of LZ1309 strain were inoculated into the CEF cells or LY294002-treated CEF cells in 6-well plates and incubated at 37°C with 5% CO2. The virus infected CEFs were collected at 24, 48, 72, 96 hpi hours post-inoculation (hpi), and a series of twofold dilutions was prepared and distributed in triplicate into 96 well plates containing the CEFs. The viral titers at each time point were calculated based on the number of pfu. And the method to detect MDV genome copy numbers was real-time quantitative PCR. After infection of MDV in CEF cells or LY294002-treated CEF cells, DNA of virus infected CEFs was prepared at 24, 48, 72, 96 hpi using the Tissue DNA Kit (Takara, Dalian,China) according to the manufacturer’s instructions. The method of real-time quantitative PCR was previously described (Baigent et al., 2005), using the Premix Ex Taq^TM^ (Probe qPCR, Takara, Dalian,China).

### Antibodies and Reagents

Rabbit antibodies against phospho-Akt (Ser473), phospho-Akt (Thr308), Akt, phospho-p85 (Tyr458), p85, phospho-GSK-3β (Ser9), GSK-3β, phospho-mTOR (Ser2448), mTOR, glyceraldehyde 3-phosphate dehydrogenase (GADPH), and inhibitors LY294002, LY303511 and wortmannin were purchased from Cell Signaling Technology (Beverly, MA, United States). Mouse anti-FLAG antibody and anti-GFP were provided from Thermo Fisher Scientific (1:1000, Thermo Fisher Scientific, Shanghai, China). Secondary infrared dye 800CW goat anti-rabbit and anti-mouse IgGs were purchased from LI-COR Biosciences (Lincoln, NE, United States). And Alexa Flour 594 was purchased from Thermo Fisher Scientific (Thermo Fisher Scientific, Shanghai, China).

### Cell Viability Assay

Cell viability was measured using the Cell Counting Kit-8 (CCK-8; Beyotime Institute of Biotechnology, Jiangsu, China). 104 CEF cells/well were seeded in a 96-well plates, incubated at 37°C for 24 h, and placed in serum-free conditions for another 1 h. Then cells were washed twice with PBS, and treated with LY294002 (5–50 mM) or 0.1% dimethyl sulfoxide (DMSO) used as vehicle control for 24 h at 37°C. Then, CCK-8 dye was added for 2 h at 37°C and the absorbance was measured at 450 nm in a Multiskan FC microplate reader (Thermo Fisher Scientific, Shanghai, China).

### Flow Cytometry

Cell apoptosis was determined by flow cytometry using the Annexin V-FITC/PI Apoptosis Detection Kit (Sigma-Aldrich, MO, United States) following the manufacturer’s instructions. CEFs (1 × 10^6^) were pretreated with LY294002 (20 μM), or 0.1% DMSO for 1 h and infected with MDV LZ1309 strain at a MOI of 0.1. At indicated time (24, 48 and 72 h),cells were washed with ice-cold PBS three times, centrifuged, suspended in 500 μL 10 × binding buffer provided by the Kit, and incubated with 10 uL Annexin V for 10 min and then with 5 μL propidium iodide (PI) for 5 min at room temperature. Cells were quantified and analyzed using a FC500 flow cytometer (Beckman Coulter, Brea, CA, United States). As controls, LY303511 (30 μM) and wortmannin (500 μM) were used to treat the CEFs prior to infect MDV, cells were collected at 72 hpi and tested the apoptotic. The data were expressed as the mean percentage of apoptotic cells based on three independent experiments.

### Western Blotting

Cells were washed with PBS and lysed by cell lysis buffer for western blotting and immunoprecipitation (Beyotime Institute of Biotechnology) on ice for 8 min. Cell lysates were centrifuged at 12,000 × *g* for 5 min and protein content in supernatant was determined using the BCA assay (Fermentas, Thermo Fisher Scientific). Total cell proteins (20 μg) were resolved by SDS-PAGE in 10% gels and transferred onto nitrocellulose membranes (Whatman, Maidstone, United Kingdom), which were blocked with 5% (w/v) bovine serum albumin (BSA) for 1 h at 37°C and then incubated with primary antibodies (1:1000, Cell Signaling Technology, MA, United States)at 4°C overnight. After rinsing three times with Tris-buffered saline/Tween 20 (2000:1,TBST), the membranes were incubated at 37°C for 1 h with secondary IRDye 800DX-conjugated antibodies (1:10,000, LI-COR Biosciences, NE, United States) diluted in TBST. Membranes were washed five times with TBST and specific signals were analyzed using an Odyssey infrared imaging system (LI-COR Biosciences). Image J software was used for quantification of the intensity of each band and results represented as with ratio of phosphorylated protein to total protein.

### Co-immunoprecipitation

To construct recombinant eukaryotic expression plasmids, the *PIK3R1* and *PIK3R2* genes encoding PI3K regulatory subunits p85α and p85β (GenBank accession numbers XM_424759.5 and XM_001233340.4, respectively), total RNA of uninfected CEF cells was extracted using the PrimeScript^TM^ RT Reagent kit (Takara, Dalian, China) according to the manufacturer’s protocol. RNA concentrations were determined using a spectrophotometer (260 nm/280 nm). *PIK3R1* and *PIK3R2* genes were generated by RT-PCR with a PrimeScript RT reagent Kit (Takara, Dalian, China). The following primers were used: *PIK3R1* forward: 5′-CCTCGAGGATGATGGCTGAAGGATATCA-3′ and reverse: 5′-CGGATCCGTCAATCGCCTCTGCTGTGCAT-3′; *PIK3R2* forward: 5′-CCTCGAGGATGGGCAGAACTGACGGATTC-3′ and reverse: 5′-CGGATCCGTCATATGGATGGTGGCTGGGA-3′; And the *meq* gene of the MDV LZ1309 strain (GenBank accession number KX966280.1) were amplified by PCR which DNA was extracted using DNA Extraction Kit (Takara, Dalian, China) from LZ1309-infected cells. The following primers were used: *meq* forward: 5′-CCAAGCTTGGATGTCAGGAGCCAGAGCC-3′ and reverse: 5′-CGGATCCTCAGGGTCTCCCGTCACCTG-3′.

The amplified fragments were inserted into eukaryotic expression vectors p3 × FLAG-CMV-10 (*meq*) and pEGFP-N1 (*PIK3R1* and *PIK3R2*). And the resultant plasmids p3 × FLAG-CMV-10 (*meq*) and pEGFP-N1 (*PIK3R1*) or p3 × FLAG-CMV-10 (*meq*) and pEGFP-N1 (*PIK3R2*) were used to transfect DF-1 cells, cells transfected with empty vectors were used as mock control. After 36 h, the expression of recombinant proteins was examined by indirect immunofluorescence and western blotting. Cell lysates were prepared as described in chapter “Flow Cytometry” and incubated with the anti-FLAG antibody or GFP-specific antibody immobilized to protein A-Sepharose beads for 2 h at 4°C. After washing with PBS, the precipitated proteins were subjected to SDS-PAGE, followed by western blotting.

### Confocal Immunofluorescence Microscopy

Transfected DF-1 cells were gently washed with cold PBS, fixed with 200 μL of 4% paraformaldehyde at room temperature for 30 min, permeabilized with 0.2% Triton X-100 for 15 min, blocked with 5% BSA at 37°C for 1 h, and incubated with the anti-FLAG primary antibody at 37°C for 1 h and then with Alexa Fluor 594-conjugated secondary antibody (1:1000, Thermo Fisher Scientific, Shanghai, China) in the dark for 1 h. After addition of DAPI staining with fluorescence quenching agent (Thermo Fisher Scientific, Shanghai, China), cell nuclei were visualized by DAPI staining. Cell monolayers were observed under a laser confocal microscope (Leica, Wetzlar, Germany).

### Statistical Analysis

The results were expressed as the mean ± SD or SEM and statistically compared by two-way ANOVA using the SPSS software (version 17.0). Differences between groups were considered significant at *p* < 0.05.

## Results

### MDV Infection Promotes Akt Phosphorylation in CEFs

**FIGURE 1:**
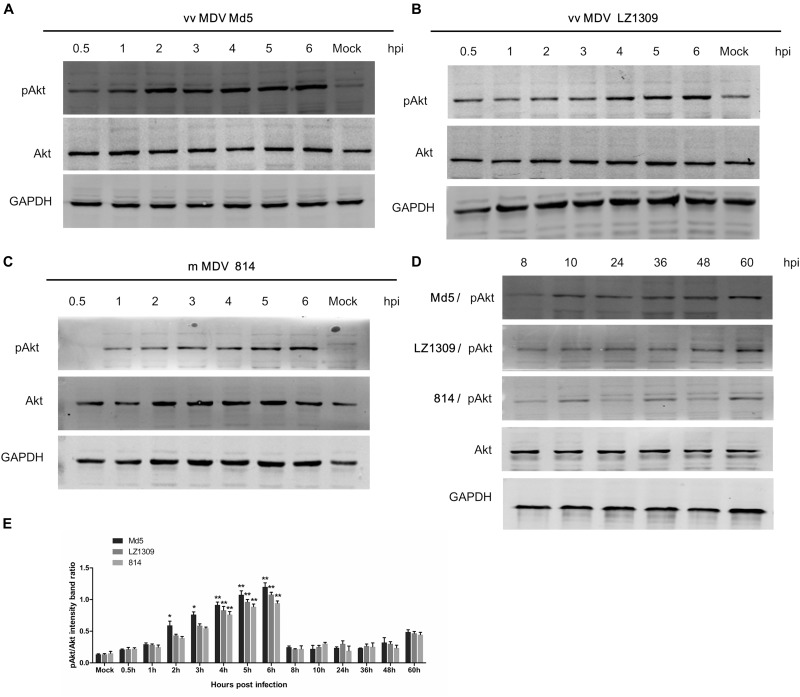
MDV infection activates Akt phosphorylation in CEFs. Serum-starved CEFs were infected with vvMDV Md5, vvMDV LZ1309, or mMDV 814 at 0.1 MOI for 0.5, 1, 2, 3, 4, 5, 6, 8, 10, 24, 36, 48, or 60 h, mock group (uninfected CEFs) and analyzed for Akt phosphorylation at Ser473 (pAkt 473) and total Akt expression by western blotting; GAPDH was used as a loading control. **(A)** vvMDV Md5 infection significantly increased Akt phosphorylation in CEFs at 2–6 hpi. **(B)** vvMDV LZ1309 infection significantly increased Akt phosphorylation in CEFs at 4–6 hpi. **(C)** mMDV 814 infection significantly increased Akt phosphorylation in CEFs at 4–6 hpi. **(D)** MDV infection reduced Akt phosphorylation in CEFs at 8–48 hpi and slightly induced it at 60 hpi. **(E)** Quantification of relative pAkt band intensities to Akt in **(A–D)**. The data represent the mean ± SD of three independent experiments. Two-way ANOVA, *^∗^P* < 0.05, *^∗∗^P* < 0.01.

To determine whether MDV could activate the PI3K/Akt pathway, CEFs were infected with MDV strains Md5, LZ1309, or 814 at 0.1 MOI and analyzed for Akt phosphorylation at different time points over 60 h post infection (hpi). The results indicated that all tested MDV strains induced Akt phosphorylation at Ser473 as early as at 0.5 hpi; the highest phosphorylation level was observed at 4–6 hpi, gradually declined at 8–48 hpi and was slightly induced again at 60 hpi (Figure 1).

### MDV Induces Akt Phosphorylation in a PI3K-Dependent Manner

**FIGURE 2:**
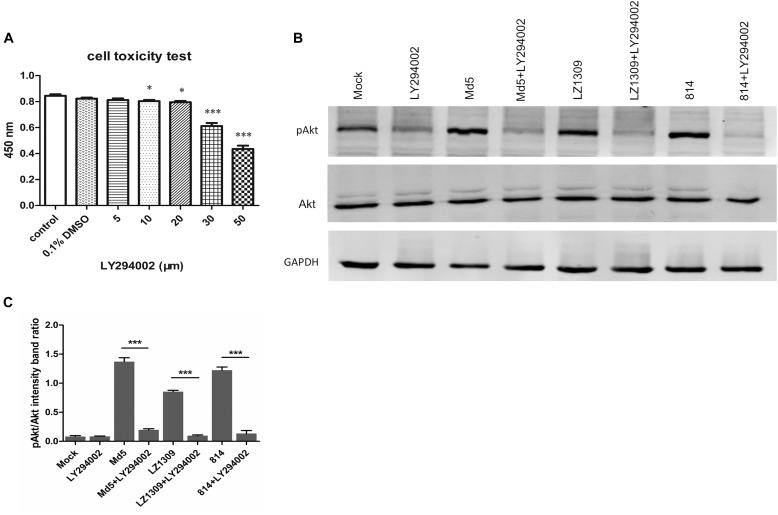
MDV infection activates Akt phosphorylation in CEFs in a PI3K-dependent manner. **(A)** Toxicity testing of PI3K-specific inhibitor LY294002. CEFs were treated with LY294002 (5–50 μM) and analyzed for survival using the CCK-8 assay. The data are expressed as the mean ± SD of three independent experiments; ^∗^*p* < 0.05, ^∗∗^*p* < 0.01, and ^∗∗∗^*p* < 0.001. **(B)** LY294002 blocked MDV-induced Akt phosphorylation. CEFs were pre-incubated with LY294002 (20 μM) for 1 h, infected with MDV Md5, LZ1309, and 814 strains at 0.1 MOI for 6 h, and cell lysates were analyzed for the expression of phospho-Akt (Ser 473), total Akt, and GAPDH by western blotting. **(C)** Quantification of relative pAkt band intensities to Akt in **(B)**. The data represent the mean ± SD of three independent experiments. One-way ANOVA, *^∗∗∗^P* < 0.001.

To verify whether the phosphorylation of Akt in MDV-infected CEFs was mediated by the upstream signaling molecule PI3K, CEFs were incubated with a specific PI3K inhibitor LY294002 prior to MDV infection. The CCK-8 assay showed that the optimum LY294002 concentration not affecting CEF viability was 20 μM (Figure 2A), which was used in further experiments. Pretreatment with LY294002 significantly reduced Akt phosphorylation in MDV-infected CEFs compared with infected cells not treated with the inhibitor (Figure 2B). These results demonstrated that MDV induced Akt phosphorylation in CEFs through PI3K activation.

### PI3K/Akt Pathway Regulates MDV Replication

**FIGURE 3:**
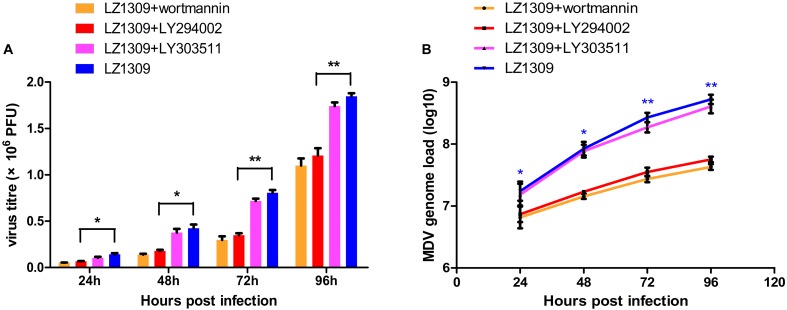
PI3K specific inhibitor LY294002 inhibits MDV replication. CEFs were pre-incubated with LY294002 (20 μM), LY303511 (30 μM) or wortmannin (500 μM) for 1 h, infected with MDV-LZ1309 at 0.1 MOI and analyzed for virus replication at 24, 48, 72, and 96 hpi. **(A)** MDV plaque quantification. **(B)** TaqMan real-time PCR detection of viral DNA. The data represent the mean ± SD of three independent experiments. One-way ANOVA, *^∗^P* < 0.05, *^∗∗^P* < 0.01.

To investigate whether activation of PI3K/Akt signaling by MDV was critical for virus replication, CEFs pretreated or not with LY294002 were infected with MDV and virus titers were determined by the plaque assay and real-time quantitative PCR at 24, 48, 72, and 96 hpi. LY294002 and wortmannin treatment groups significantly reduced MDV replication and viral DNA synthesis compared to untreated cells or LY303511-treated cells (Figure 3), indicating that the activation of PI3K/Akt signaling by MDV plays a role in the viral replication cycle.

### Inhibition of the PI3K/Akt Pathway Activated by MDV Promotes Apoptosis

**FIGURE 4:**
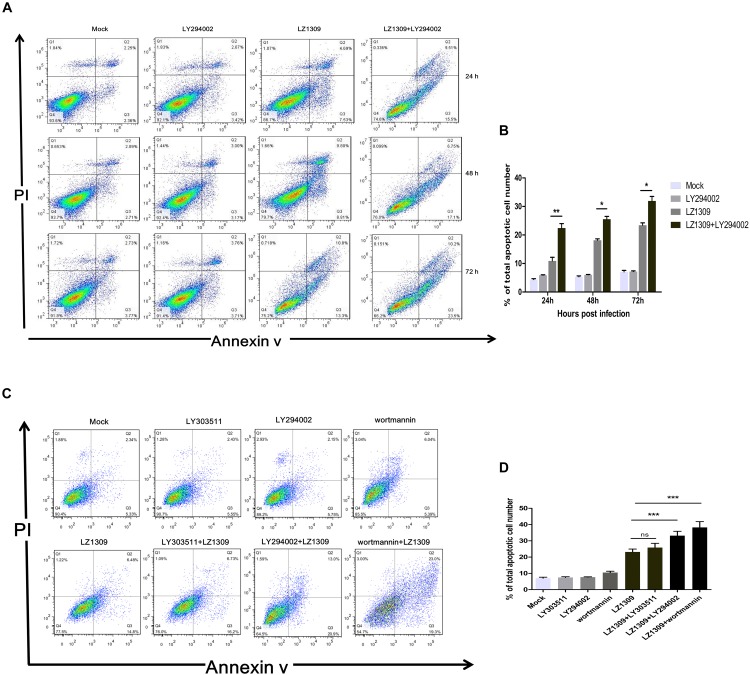
Inhibition of PI3K activity results in the apoptosis of MDV-infected CEF cells. **(A)** CEFs were pre-treated or not with LY294002 (20 μM), for 1 h, infected with MDV-LZ1309, and analyzed for apoptosis at 24, 48, and 72 hpi by flow cytometry after Annexin V-FITC/PI double staining. **(B)** Quantification of the numbers of apoptotic cells of **(A)**. **(C)** CEFs were pre-treated or not with LY294002 (20 μM), LY303511 (30 μM) or wortmannin (500 μM) for 1 h, infected with MDV-LZ1309, and analyzed for apoptosis at 72 hpi by flow cytometry after Annexin V-FITC/PI double staining; **(D)** Quantification of the numbers of apoptotic cells of **(C)**. The data represent the mean ± SD of three independent experiments. Two-way ANOVA, *^∗^P* < 0.05, *^∗∗^P* < 0.01, *^∗∗∗^P* < 0.001.

PI3K/Akt-mediated phosphorylation of cytoplasmic proteins can delay apoptosis, which is beneficial for viral reproduction. To examine whether inhibition of PI3K/Akt signaling in MDV-infected cells could induce apoptosis, CEFs were pretreated or not with LY294002, LY303511 or wortmannin before MDV infection and analyzed for the apoptotic rate by flow cytometry. Compared with apoptosis of mock group (0.1% DMSO; 2.36, 2.71, and 3.77%), apoptosis of LZ1309 strain-infected CEFs gradually increased with the time post infection: to 7.53, 8.81, and 13.3% at 24, 48, and 72 hpi, respectively, and LY294002 pretreatment further aggravated the effect as evidenced by significantly elevated percentage of apoptotic cells (15.5, 17.1 and 23.5, respectively) (Figure 4). The apoptotic cells of wortmannin pretreatment increased significantly compared with untreated cells, while found no difference between LY303511 pretreatment and LZ1309-infected group at 72 hpi. These results indicated that inhibition of PI3K/Akt signaling promoted apoptosis of MDV-infected CEFs.

### MDV Infection Induces Phosphorylation of Signaling Proteins Upstream and Downstream of Akt

**FIGURE 5:**
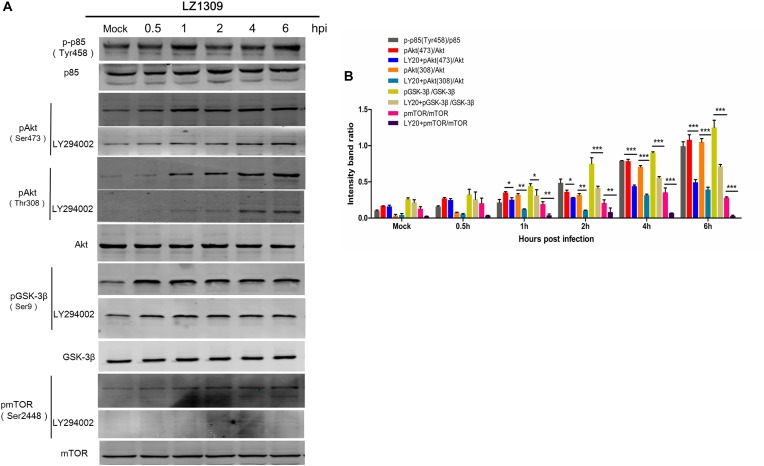
MDV induces phosphorylation of signaling proteins involved in the PI3K/Akt pathway. **(A)** CEFs cells were pre-incubated or not with LY294002 (20 μM) for 1 h and infected with MDV-LZ1309 at 0.1 MOI. Cells were collected at 0, 0.5, 1, 2, 4, and 6 hpi and cell lysates examined for phospho-p85 (Tyr458), total p85, phospho-Akt (Ser473), phospho-Akt (Thr308), total Akt, phospho-GSK-3β (Ser9), total GSK-3β, phospho-mTOR (Ser2448), total mTOR, and GAPDH by western blotting. **(B)** Quantification of relative phosphorylated protein band intensities to total protein in **(A)**. The data represent the mean ± SD of three independent experiments. Two-way ANOVA, *^∗^P < 0.05.*

To comprehensively explore the activation of PI3K/Akt signaling during MDV replication, we examined the phosphorylation level of other signaling proteins acting in the PI3K/Akt pathway. For this, CEFs pretreated or not with the PI3K inhibitor LY294002 for 1 h were infected with MDV LZ1309 and analyzed for protein phosphorylation at 0.5, 1, 2, 4, and 6 hpi by western blotting. The results showed that MDV infection of CEFs increased the phosphorylation of the PI3K regulatory subunit p85 (Tyr458) upstream of Akt as well as that Akt (Ser473), Akt (Thr308), GSK-3β (Ser9) and mTOR (Ser2448), whereas LY294002 decreased Akt, GSK-3β and mTOR phosphorylation (Figure 5). These results showed that MDV infection upregulated the phosphorylation of key signaling molecules in the PI3K/Akt pathway, including those upstream and downstream of Akt. As GSK-3β and mTOR are known to be closely involved in apoptosis and oncogenesis (Mancinelli et al., 2017; Guri et al., 2018), our findings suggest that activation of the PI3K/Akt pathway plays an important role in the establishment and pathogenesis of MDV infection.

### Interaction of Viral Protein Meq With the p85 Subunit of PI3K

**FIGURE 6:**
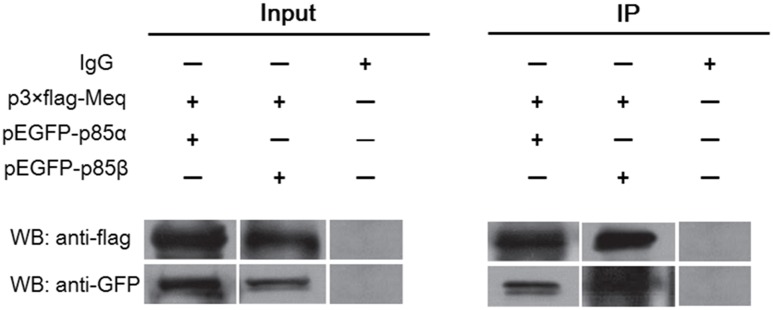
The Meq protein of MDV interacts with PI3K regulatory subunits p85α and p85β. Lysates of DF-1 cells overexpressing FLAG-tagged Meq together with EGFP-tagged p85α or EGFP-tagged p85β were subjected to co-immunoprecipitation using FLAG-specific and GFP-specific antibodies, respectively. WB, western blotting; IP, immunoprecipitation.

Similar to the LMP1 protein of EBV, the Meq protein of MDV is directly involved in virus-induced oncogenesis. As Meq contains five PXXP motifs potentially interacting with the p85 subunit of PI3K, we examined whether Meq could bind p85. For this, cellular lysates from DF-1 cells co-transfected with eukaryotic expression plasmids p3 × FLAG-Meq and pEGFP-p85α or pEGFP-p85β were subjected to immunoprecipitation with anti-FLAG or anti-GFP antibodies and analyzed by western blotting. The results showed that Meq was able to pull-down both p85α and p85β subunits (Figure 6), indicating that Meq directly interacted with PI3K and suggesting a possible role of this MDV protein in the activation of the PI3K/Akt pathway in host cells.

### Colocalization of Meq and p85 in DF-1 Cells

**FIGURE 7:**
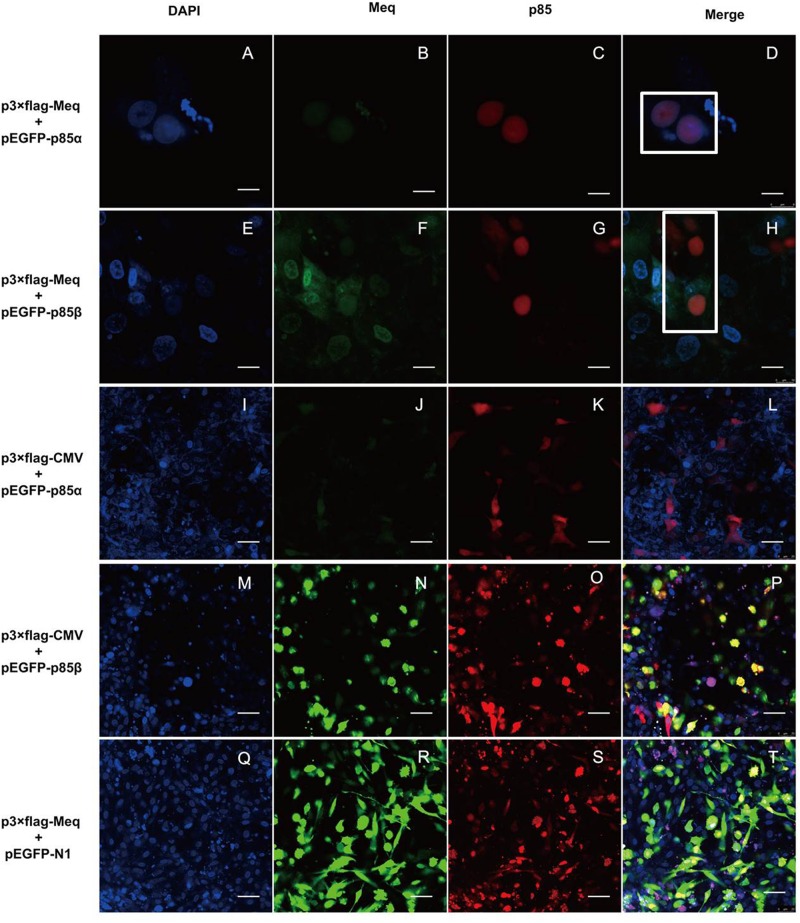
The Meq protein colocalizes with p85α and p85β subunits of PI3K in DF-1 cells. DF-1 cells were transfected with eukaryotic expression plasmids for 24 h and analyzed by laser scanning confocal microscopy after immunostaining with anti-FLAG and Alex Flour 546-conjugated antibodies (red); EGFP-tagged p85 subunits are seen green and DAPI-stained nuclei are blue. **(A–D)** DF-1 cells transfected with p3 × FLAG-Meq and pEGFP-p85α plasmids; scale bars, 8 μm. **(E–H)** DF-1 cells transfected with p3 × FLAG-Meq and pEGFP-p85β plasmids; scale bars, 10 μm. **(I–L)** DF-1 cells transfected with empty vector p3 × FLAG-CMV-10 and the pEGFP-p85α plasmid; scale bars, 25 μm. **(M–P)** DF-1 cells transfected with empty vector p3 × FLAG-CMV-10 and the pEGFP-p85β plasmid; scale bars, 25 μm. **(Q–T)** DF-1 cells transfected with the p3 × FLAG-Meq plasmid and empty vector pEGFP-N1; scale bars, 25 μm. White box indicates intracellular protein colocalization.

To further verify the interaction of Meq with p85α and p85β subunits of PI3K, we examined their localization in DF-1 cells co-expressing Meq and p85 by confocal microscopy. The results showed that Meq colocalized with p85α or p85β in cells co-transfected with Meq and p85 expression plasmids, but not in those expressing Meq or p85 alone (Figure 7), further confirming that the Meq protein of MDV interacted with the p85α and p85β subunits of PI3K in chicken fibroblasts.

### Meq Activates PI3K/Akt Signaling in DF-1 Cells

**FIGURE 8:**
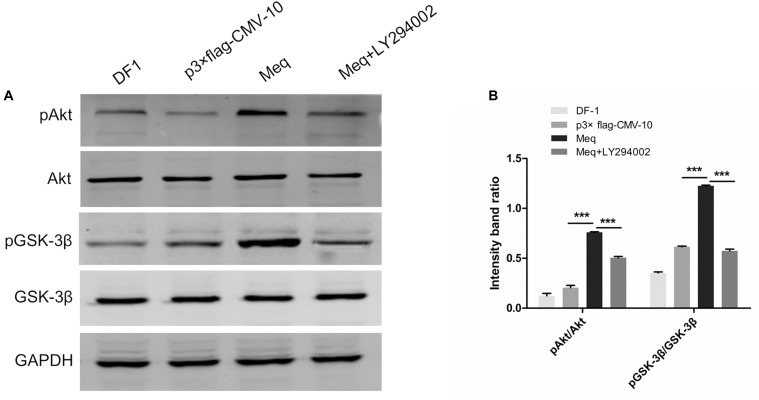
The MDV Meq protein activates PI3K/Akt signaling. **(A)** DF-1 cells were pre-incubated or not with LY294002 (20 μM) for 1 h, transfected with the p3 × FLAG-Meq plasmid or empty vector p3 × FLAG-CMV, and cell lysates were analyzed 36 h post transfection for phospho-Akt (Ser 473), total Akt, phospho-GSK-3β, total GSK-3β, and GAPDH by western blotting. **(B)** Quantification of relative phosphorylated protein band intensities to total protein in **(A)**. The data represent the mean ± SD of three independent experiments. One-way ANOVA, ^∗∗∗^*P* < 0.001.

As the results described above indicate that Meq binds to p85α and p85, we next examined whether this interaction might be involved in the activation of PI3K/Akt signaling during MDV infection. For this, DF-1 cells were transfected with the Meq expression plasmid for 36 h, treated or not with LY294002, and analyzed for the phosphorylation of Akt and GSK-3β. The results showed that Meq induced Akt and GSK-3β phosphorylation (Figure 8), suggesting that MDV can activate the PI3K/Akt pathway through Meq interaction with the p85 regulatory subunit of PI3K.

## Discussion

During infection of host cells, many viruses activate intracellular PI3K/Akt signaling, as this pathway, which plays a critical regulatory role in cell growth and apoptosis, may support the survival of host cells during viral replication. However, it has been unknown whether MDV induces PI3K/Akt signaling in infected cells. In this study, we showed that MDV infection enhanced Akt phosphorylation in a PI3K-dependent manner at the early stage of infection and that Akt inhibition decreases MDV replication while increasing apoptosis, suggesting that the activation of the PI3K/Akt pathway supports the infectious cycle of MDV through maintaining survival of host cells. By addressing the underlying mechanism, we found that MDV activated PI3K/Akt signaling through interaction between its protein Meq and PI3K regulatory subunits p85α and p85β. Cumulatively, these results suggest that MDV hijacks cell signaling machinery to maintain and propagate its infection in the host.

Our data are consistent with previous findings that many viruses activate the PI3K/Akt signaling pathway in the early stages of infection to support viral replication. Activated Akt is involved in the inhibition of apoptosis and induction of cell survival through phosphorylation of its downstream targets such as BAD, p53, and FOXO1 (Rodgers et al., 2017); consequently, virus activation of Akt ensures viral replication and spread through maintenance of cell survival. Influenza A virus can increase the expression of phosphorylated Akt protein in A549 cells at 6–12 hpi (Ehrhardt et al., 2007), whereas inhibition of Akt decreases viral entry and intracellular replication (Hirata et al., 2014). Activation of PI3K/Akt resulting in support of virus replication was observed for avian leukosis virus (Feng et al., 2011), whereas Newcastle disease virus also inhibited cell apoptosis and enhanced autophagy (Kang et al., 2017) MDV activation of PI3K/Akt signaling also occurred early in the infection; however, it was observed later compared to RNA viruses such as Newcastle disease virus and avian reovirus and leukosis virus, probably because MDV transmission requires cell-to-cell contact (Favoreel et al., 2005), which delays PI3K/Akt activation. Blocking PI3K activity with the specific inhibitor LY294002, we found that LY294002 treatment inhibited MDV replication and promoted apoptosis in the infected cells (Figures 3, 4). This indicates that activation of the PI3K/Akt pathway is likely to support MDV propagation through inhibiting apoptosis in infected cells. Moreover, previous study have showed that LY294002 decreased the expression of p-IκBα, p-Stat3 and p-p70S6 kinase results in an inflammatory response and delayed apoptosis in avian reovirus-infected cells (Lin et al., 2010a,b). And EBV-transformed B-cells were particularly dependent on constitutive NF-κB activity through LMP1, and rapidly undergo apoptosis upon NF-κB blockade (Ersing et al., 2013). Further investigation is required to understand whether LY294002 treatment affect the ability of Meq to induce NFκB pathway.

Viruses employ different strategies to activate the PI3K/Akt pathway in infected cells. One is the interaction between viral proteins and PI3K, Akt, or mTOR, leading to abnormal activation of PI3K/Akt signaling (Dunn and Connor, 2012). It was shown that viruses expressing proteins with PXXP and YXXXM/YXXM motifs could bind SH3 and SH2 domains of the p85 subunit, thereby activating the PI3K/Akt pathway to facilitate virus invasion and replication (Kay et al., 2000; Zhang et al., 2002; Street et al., 2004). The amino acid sequence of the VP11/12 protein of HSV-1 contains an YXXM motif (Y519, YXXM) which interacts with p85, leading to PI3K/Akt activation (Wagner and Smiley, 2011; Strunk et al., 2013). The ORF12 protein of Varicella zoster virus has high homology with the VP11/12 protein of HSV-1 and contains a YXXM motif (Y249), which binds to the p85 subunit and activates PI3K to regulate cell cycle progression (Liu and Cohen, 2013). The NS1 protein of influenza A virus binds to p85β through its PXXP motif (residues 164–167) and activates the PI3K/Akt pathway, thus supporting virus replication by inhibiting virus-induced apoptosis (Shin et al., 2007a). Infectious bursal disease virus expresses the VP5 protein containing three PXXP motifs (residues 76–79, 102–105, and 145–148); the protein interacts with the p85α but not p85β subunit of PI3K and induces PI3K/Akt activation, resulting in the suppression of apoptosis and enhancement of virus proliferation (Wei et al., 2011). In this study, we observed that the Meq protein of MDV, which has four PXXP motifs (residues 11–14, 40–43, 122–125, 140–143, and 157–160), could bind both PI3K regulatory subunits, p85α and p85β, which led to Akt activation; however, it remains to be clarified which of the SH3-binding motifs are responsible for Meq interaction with p85α and p85β and contribute to PI3K/Akt activation. To provide further insights into the role of Meq in PI3K/Akt activation and MDV infection of host cells, we plan to perform sequential deletion mutagenesis of the PXXP motifs in Meq and analyze binding of the mutant Meq proteins to p85α and p85β as well as to construct Meq-deficient MDV strains and investigate infection rates and PI3K/Akt activation in host cells.

PI3K/Akt signaling is stimulated in several herpesvirus-associated lymphoproliferative disorders, including EBV-induced T-cell lymphocytosis, Kaposi’s sarcoma, Hodgkin’s lymphoma, and nasopharyngeal carcinoma (Benetti and Roizman, 2006; Alsayed et al., 2008; Chen, 2012). K1 protein, one of Kaposi’s sarcoma virus oncogenes, can activate the PI3K/Akt pathway in Kaposi’s sarcoma and primary effusion lymphoma (Tomlinson and Damania, 2004; Bhatt and Damania, 2013). Our results show that MDV activation of the PI3K/Akt pathway through Meq interaction with p85α and p85β results in the phosphorylation of GSK-3β and mTOR associated with malignant transformation (Manning and Toker, 2017), which is consistent with previous findings that Meq inhibits apoptosis and is an MDV oncogene (Liu et al., 1998; Silva et al., 2010). Further studies should be performed to determine whether the activation of the PI3K/Akt pathway by MDV through Meq is associated with tumorigenesis in MD.

## Conclusion

In conclusion, our data indicate that MDV activates the PI3K/Akt signaling pathway in infected host cells through interaction of its protein Meq with p85 regulatory subunits of PI3K, which promotes viral replication. These findings should contribute to elucidation of the molecular mechanism underlying MD pathogenesis and could potentially promote the development of novel approaches to prevent MDV spread in poultry. Although the viral Meq protein activates the PI3K/Akt pathway during MDV infection, further studies are therefore required to determine the relationship between this pathway and MDV tumorigenic mechanism.

## Author Contributions

RC conceived the study and wrote the paper. HL and JZ designed and performed the experiments, and analyzed the data. MH and QL provided technical assistance and prepared the figures. FL revised the manuscript. All authors reviewed the results and approved the final version of the manuscript.

## Conflict of Interest Statement

The authors declare that the research was conducted in the absence of any commercial or financial relationships that could be construed as a potential conflict of interest.
